# Acute soft head syndrome in a teenager with sickle cell anemia: A case report

**DOI:** 10.1002/ccr3.8174

**Published:** 2023-11-06

**Authors:** Ng'weina F. Magitta, Francisca B. Komanya, Baraka O. Alphonce, Mbelwa D. Bitesigilwe, Emmanuel M. Sindato, John R. Meda

**Affiliations:** ^1^ Department of Internal Medicine, School of Medicine & Dentistry University of Dodoma Dodoma Tanzania; ^2^ Department of Biochemistry & Clinical Pharmacology, Mbeya College of Health & Allied Sciences University of Dar es Salaam Mbeya Tanzania; ^3^ Department of Internal Medicine Benjamin Mkapa Hospital Dodoma Tanzania; ^4^ Department of Radiology & Imaging Benjamin Mkapa Hospital Dodoma Tanzania

**Keywords:** acute soft head syndrome, bone infarction, extramedullary hematopoiesis, sickle cell anemia, subgaleal haematoma

## Abstract

**Key Clinical Message:**

Sickle cell disease (SCD) rarely presents with acute soft head syndrome (ASHS) often posing a diagnostic dilemma. Recovery is typically spontaneous, however, in the context of lack of awareness and limited brain imaging it could potentially lead to poor outcome.

**Abstract:**

ASHS is a rare complication of SCD, invariably occurring near puberty with hitherto elusive pathogenic mechanisms. ASHS often resolves spontaneously on conservative management, however, lack of awareness in the context of limited access to brain imaging could pose diagnostic challenges resulting in inappropriate management and untoward outcome. We present a case of a teenager who presented with subtle symptoms for which the diagnosis of sickle cell anemia (SCA) was delayed until he developed ASHS. LTM was a 16 years old boy with a history of recurrent joints pain since the age of 6 years, with a family history of SCA, but had initial negative sickling test. He presented with episodes of multiple joints pain, unprovoked scalp and left orbital swelling, low‐grade fever and mild headache without any evidence for bleeding diathesis. The diagnosis of SCA was confirmed by hemoglobin electrophoresis. Computed tomography (CT) scan of the head revealed subgaleal heamatoma (SGH) and intraorbital haematoma without intracranial hemorrhage (ICH). He was managed conservatively with analgesics and hydration together with antibiotics for associated sepsis with complete resolution of clinical symptoms within 2 weeks. This case represents a rare scenario for a relatively mild SCA phenotype presenting with ASHS whose diagnosis poses an enigma in the resource‐limited contex. It is therefore, prudent to recognize ASHS to avoid judicious interventions which could potentially result in untoward clinical outcome.

## Graphical abstract

Acute soft head syndrome in sickel cell disease

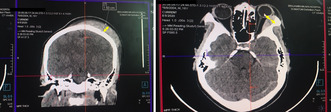

## 1 BACKGROUND

Sickle cell disease (SCD) is a group of autosomal recessive haemoglobinopathies characterized by propensity towards polymerization and sickling of red blood cells (RBCs) at low partial pressure of oxygen.^1^, ^2^, ^3^ The most severe clinical phenotype, termed sickle cell anemia (SCA, MIM#603903) results from homozygosity of the mutant gene.^1^, ^2^, ^3^ Sickle cell trait encompasses heterozygous genotypes of SCD, which do not usually trigger a clinical phenotype except under extreme hypoxic conditions. The disease was first described by Harrick in 1910.^4^ SCD results from a single base‐pair missense point mutation of the β globin gene on chromosome 11p15. There are a plethora of genetic variants, however, HBB‐B^s^ is the most common and clinically significant genotype, [HBB; c.20 T > A, p.Glu6Val; OMIM: 141900 (HBB‐β^S^)].^1^, ^2^ The polymerization of mutant β‐globin chains and consequent sickling of RBCs results in frequent, multiple vaso‐occlusive crises (VOCs) which together with other pathogenic insults are responsible for the pleotropic manifestations and complications in multiple organs.^2^, ^3^, ^5^

The prevalence of SCD is highest in the tropics particularly in Sub‐Saharan Africa (SSA), the Mediterranean, Southeast Asia, and India where it is evolutionary known to confer protection and survival advantage in areas endemic for *P. falciparum* malaria.^1^, ^6^ The current Global Burden of Disease, Injuries and Risk Factors (GBD) study 2021 report indicate the rising trend in the prevalence of SCD in the last two decades largely attributed to the increasing birth rates in SSA and the Caribbean.^7^ Moreover, GBD 2021 report estimates the global increase by 13.7% and 41.4% of children born with SCA and the number of people living with SCA, respectively.^7^ Notably, SCA cause‐specific mortality has increased by 11‐fold, ranking SCA as the 12th cause of mortality globally.^7^

Acute soft head syndrome (ASHS) is a rare complication of SCA, which manifest with a diffuse or localized swelling on the head as a result of extravasation of blood and formation of hematoma beneath the galeal aponeurotic layer of the scalp.^8^, ^9^, ^10^ In the majority of patients, ASHS is often accompanied by epidural hematoma.^8^, ^9^, ^11^ The pathogenesis of ASHS remains partly elucidated, with several postulates. Firstly, it is believed that hypoxic response in SCA triggers sustained extramedullary hematopoiesis (EMH) in the skull bones resulting in the thinning and weakening of the cortical matrices with propensity towards fragility.^12^, ^13^ Secondly, hypoxia triggers angiogenic responses, which results in the formation of fragile local vascular beds, which act in tandem, under the context of increased cardiac output to precipitate bone breakage and extravasation of blood into the subgaleal space.^14^ Thirdly, repeated VOCs could lead to multiple, but subtle microinfarction, which over time results in altered bone and periosteal structures, bone thinning and local vessel wall necrosis leading to non‐traumatic blood extravasation to subgaleal and epidural spaces.^11^, ^15^

The diagnosis of ASHS is largely clinical. However, imaging using computed tomography (CT) scan is prudent which typically shows evidence for EMH as demonstrated by diffuse widening of the diploic space in the skull and thinning of the inner cortex due to infarction.^16^ Importantly, CT scan also serves to rule out the coexistent intracranial hemorrhage (ICH) in patients with ASHS. If obvious abnormalities are not evident, it is imperative to recognize that the extrusion of blood in the subgaleal or epidural space can potentially result in the micro fracture of the inner table.^16^

The management of ASHS is typically conservative with analgesics and good hydration using intravenous crystalloids.^8^, ^9^, ^11^ It is often prudent to administer empirical wide‐spectra antibiotics for any clinical suspicion for infection. However, as seemingly tempting, aspiration is not usually indicated unless infection is suspected based on the presence of marked features of local inflammation or marked markers for systemic inflammatory response syndrome (SIRS). Conversely, surgical intervention is indicated in the absence of spontaneous resolution or when coexisting epidural hematoma is demonstrated on a CT scan image. Inadvertently, although SGH typically resolve spontaneously, patients are at an increased risk of recurrence of both SGH and subdural hematoma. Thus, secondary prevention approaches including improved general SCA care and increased vigilance for clinical suspicion of ICH should be undertaken. Theoretically, strategies to retard EMH using folic acid supplementation and early initiation of hydroxyurea are likely to prevent ASHS.^3^, ^17^, ^18^

## 2 CASE PRESENTATION

### 2.1 Demographic details and medical history

LTM was a 16‐year‐old boy, a primary school student. He presented with a history of generalized joint pains for 4 days, which was of gradual onset, nonmigratory, persistent and associated with general body malaise, and low‐grade fever. There was no history of joint swelling, inability to use the limbs. Moreover, the patient had reported several episodes of a similar nature for the past 10 years, with an average of 5–7 episodes per year, with at least three episodes requiring hospitalization which typically resolved on conservative management with analgesics and hydration, without blood transfusion.

On the fourth day, the patient noticed a small painless swelling on the left side of the head which started spontaneously, of which within 8 h progressed extending towards the back of the head and forward to involve the left eye. The swelling was associated with mild generalized headache and low‐grade fever. There was no history of visual disturbances, swelling on other body parts, neck stiffness, convulsion, confusion, loss of consciousness, bleeding per ear, nose or throat (ENT) or projectile vomiting. The patient denied a history of recent head trauma, easy bruising, gum bleeding or use of anticoagulants.

The review of other systems was noncontributory with past medical history, which was unremarkable for blood transfusion, as well as food or drug allergy. He tested negative for sickling test at a local secondary care facility. He was the last born in a family of six siblings, grade seven primary school pupil, with one of his sibling diagnosed to have SCA at the age of 2 years, with regular clinic visits. There was no known family history of bleeding diathesis.

### 2.2 Clinical findings

**FIGURE 1:**
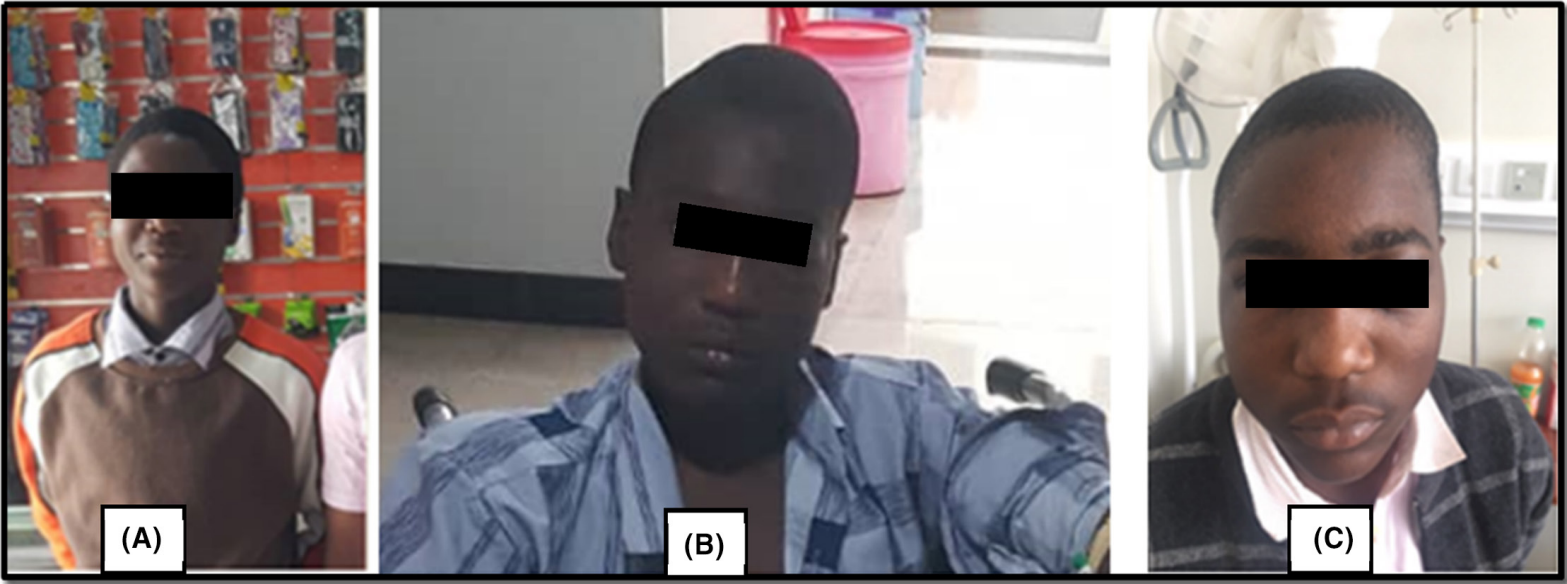
Photographs of the patient showing images. (A) Before the illness; (B) On admission; (C) Four days post‐admission.

The patient was fully conscious, with scalp and left periorbital edema, febrile (38.2°C), not pale, not dyspneic, not jaundiced, not cyanosed, and without lower limbs edema. He had stable vitals with a blood pressure of 110/80 mmHg, pulse rate of 90 beats per minute, respiratory rate of 20 breaths per minute, and 98% oxygen saturation on room air. The local examination revealed a left parietal swelling extending to the left occipital and frontal region, about 12 cm x 5 cm with normal overlying skin. The swelling was fluctuant, however, neither warm nor tender. The examination of the eyes was essentially normal except for a notable non‐tender, periorbital swelling with normal skin warmth (Figures 1A–C). ENT examination was essentially normal. There was no any obvious joint swelling, deformity or tenderness and all joints exhibited normal range of motion.

The patient was fully conscious, with good orientation, coherent and fluent speech and language and intact short and long‐term memory. All cranial nerves were intact and there were no signs of meningeal irritation. The motor and sensory modalities were intact in both upper and lower limbs, with normal coordination, gait and balance. The examination of the spine and the rest of the systems were essentially normal.

### 2.3 Diagnostic assessment

#### 2.3.1 Enhanced head CT scan

**FIGURE 2:**
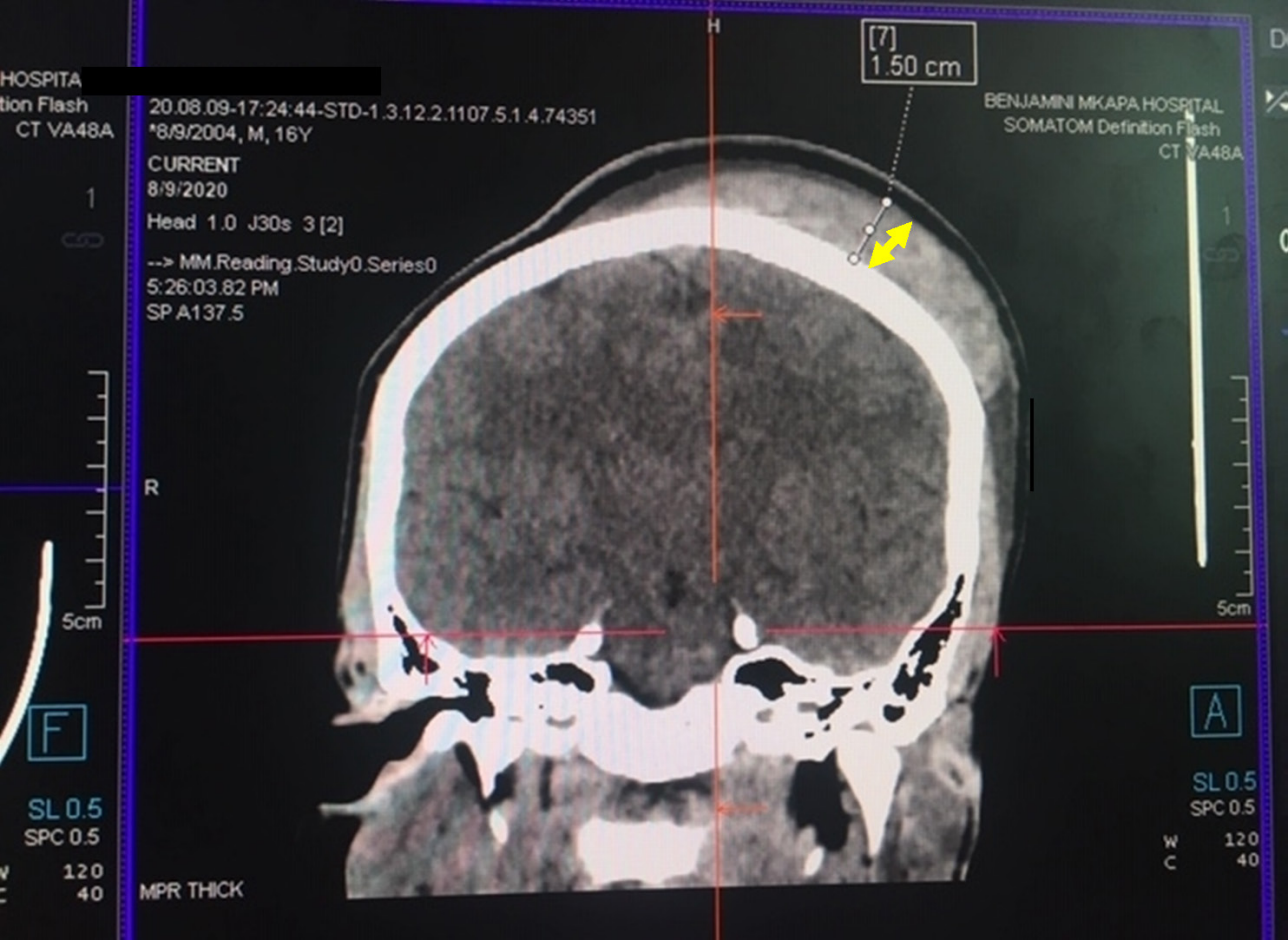
Enhanced head coronal view CT scan showing subgaleal hematoma measuring 1.5 cm (yellow arrow) overlying parietal bone with intact calvarium and normal brain parenchyma.

**FIGURE 3:**
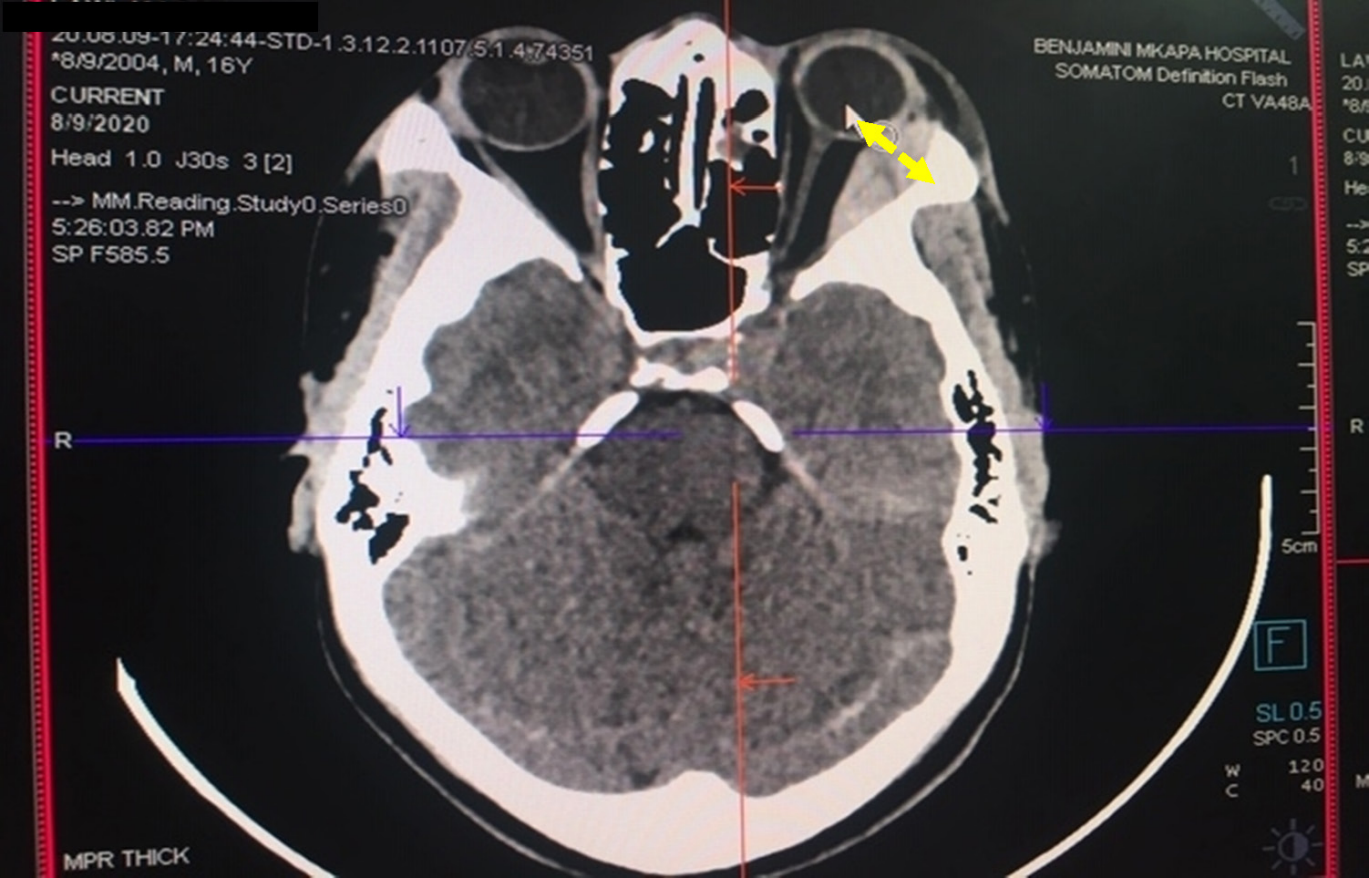
Enhanced head CT scan axial view showing left orbital hematoma with orbital pressure exertion (yellow arrow) and intact calvarium and normal brain parenchyma.

The patient underwent enhanced head CT scan imaging on the first day of the admission. The CT scan imaging revealed a hyper‐dense scalp lesion extending from the midline towards the left half of the skull covering the left parietal, left frontal and left occipital bones measuring 12 cm x 9 cm in the antero‐posterior and medio‐lateral dimensions respectively (vide infra, Figure 2). Moreover, another confined hyper‐dense lesion measuring 2.96 cm x 1.22 cm was observed in the supero‐lateral half of the left orbit with mild pressure exertion on the left orbital contents (Figure 3). The two images were indicative of features consistent with acute scalp hematoma on the left half of the skull and periorbital hematoma on the left eye.

#### 2.3.2 Laboratory investigations

The patient underwent assessment for hematological profile and diagnostic workup for SCD based on a sickling test and capillary Hb electrophoresis. He was further assessed for potential infections, liver injury and bleeding diathesis based on complete blood counts, liver enzymes, bilirubin, and bleeding indices (Table 1). The results showed normal bleeding indices and significantly detectable HbS component confirming the diagnosis of SCA suggestive of HbS homozygosity. The hemolytic markers, which included reticulocyte counts and serum lactate dehydrogenase (LDH) were significantly elevated. The liver enzymes that is,. alanine aminotransferase (ALT) and aspartate aminotransferase (AST) were significantly elevated to six and three times the upper limit of normal (ULN) respectively (vide infra, Table 1). On the second week, the liver enzymes, both ALT and AST were detectable within the normal limits.

**TABLE 1:** Baseline hematological profile, coagulopathy workup, and Hb electrophoresis.

Laboratory parameter	Normal range	Test, day 1	Test, day 4
Complete blood counts			
White blood cells	(3–11) x 10^3/μL	18	10
Neutrophils	(27–72)%	70	59.1
Red blood cells	(3–7) x 10^3/μL	3.5	3.5
Hemoglobin	(8–17) g/dl	9.8	6.4
Hematocrit	(26–50) %	29.6	20.4
Mean corpuscular volume	(86–110) fL	73.2	74.6
Mean corpuscular hemoglobin	(26–38) pg	24.3	24
Mean corpuscular Hb concentration	(31–37) g/dL	33.1	31.4
Red cell distribution	(11–16)%	21.3	22
Platelets	(150–340) x 10^3μL	151	226
Other markers			
Reticulocytes	(0.5–2.5)%	4.90	13.7
Lactate dehydrogenase	100–340 U/L	408	670
Liver enzymes & bilirubin			
Total bilirubin	(0–1.2) mg/dL	0.17	1.94
Direct bilirubin	(0–0.3) mg/dL	0.78	0.97
Alanine aminotransferase	(2–33) U/L	43.9	196
Aspartate aminotransferase	(15–37) U/L	79.1	120.4
Bleeding indices			
aPTT	30–40 sec	34	
PT	11–14 sec	12	
INR	0.8–1.1	1	
Hb electrophoresis			
HbA2	(2–3)%	0.04%	
HbF	(0.8–2)%	19.50%	
HbS	0%	80.91%	

Abbreviations: aPTT, activated partial thromboplastin time; HbA2, adult hemoglobin A2 isotype; HbF, oetal hemoglobin; and HbS, sickle hemoglobin; INR, international normalized ratio; PT, prothrombin time.

#### 2.3.3 Abdominal ultrasonography

The patient underwent abdominal ultrasonography imaging after being noted to have elevated liver enzymes. The report showed a convoluted spleen and a normal liver, both in size and architecture. The gallbladder had a normal wall architecture containing bilious sludge with normal hepatobiliary architecture, without evidence for the dilatation of the common bile duct.

### 2.4 Therapeutic intervention

The screening for SCD was done using sickling test and further confirmed by Hb electrophoresis. Moreover, the diagnosis of ASHS was reached on clinical grounds and further confirmed by a head CT scan imaging, which revealed SGH together with periorbital hematoma, without evidence for ICH. In addition, the patient had marked leukocytosis, elevated conjugated hyperbilirubinemia and elevated liver enzymes (ALT>AST) in the context of gallbladder bilious sludge without evidence of biliary obstruction. Therefore, the diagnosis of cholestasis secondary to sepsis and acute cholecystitis were entertained. The patient was managed with intravenous ceftriaxone 2 g daily for 5 days, intravenous acetaminophen 1 g 8 hourly for 24 h followed by oral intake of the same dosage along with intravenous administration of 2 L of 0.9% saline within the initial 24 h. On discharge, the patient was initiated on a long‐term therapy for SCA with supplemental folic acid 5 mg daily and hydroxyurea 1000 mg daily orally under regular SCD clinic follow up at the same facility.

### 2.5 Follow up and outcomes

**FIGURE 4:**
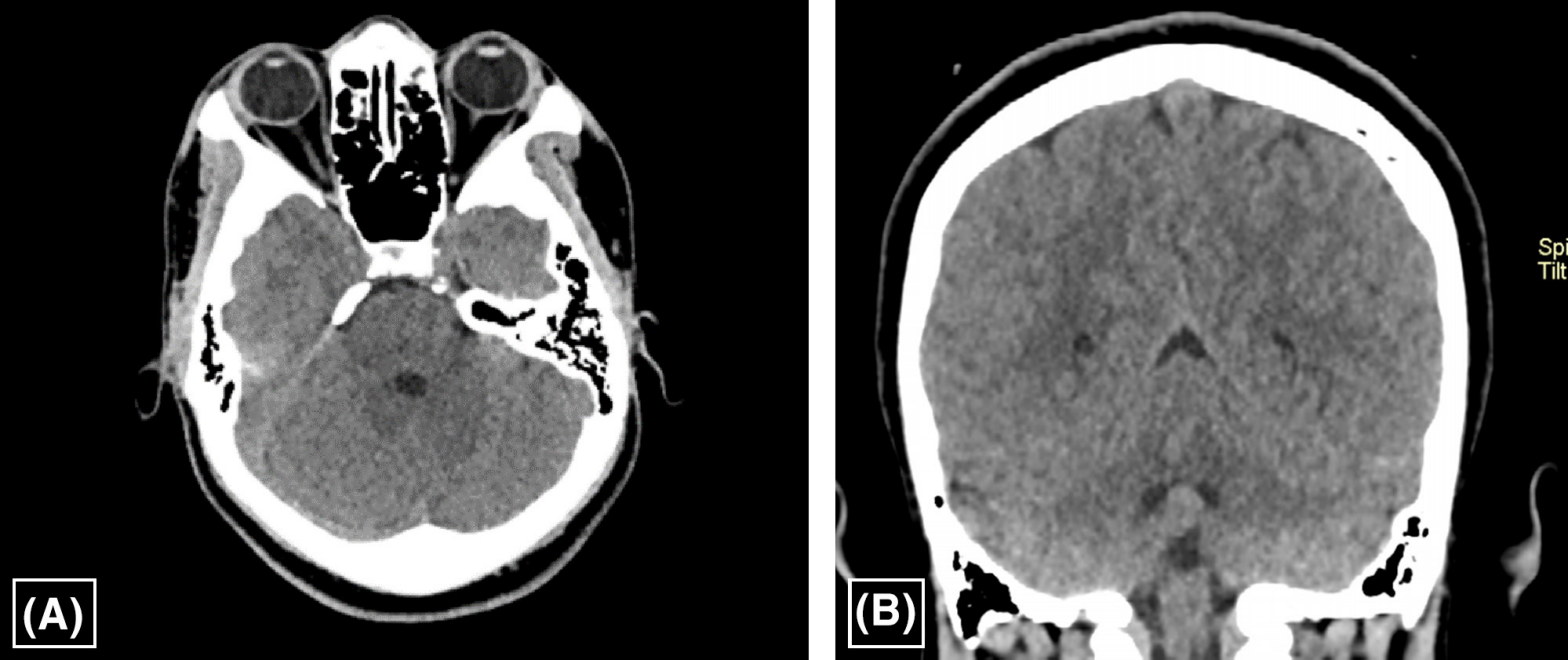
(A) Non‐enhanced axial brain CT at the level of orbits, images show: Completely resolved subperiosteal mass‐like lesion in the left retrobulbar‐extraconal region which is compatible with resolved intraorbital hematoma. (B) Non‐enhanced coronal brain CT at the level of the cerebellum, images show: Completely resolved right extra‐calvarial temporo‐parietal cephalohematoma, with no underlying skull fractures.

The patient had spontaneous recovery on conservative therapy consisting of analgesics, antibiotics and fluid administration. By the second week, the patient had complete resolution of symptoms, and the liver enzymes were detectable within the normal limits. The follow up brain CT at 2 months showed complete radiological resolution (Figures 4A, B). The patient was then followed up for 3 years without any reported episodes of similar symptoms and signs nor any neurological sequelae. He is currently attending regular SCD clinics scheduled 3‐monthly on folic acid supplementation and oral hydroxyurea. There are no reported complication or hospitalization for the last 3 years, except for minor episodes of VOCs manageable by analgesics and hydration.

## 3 DISCUSSION

ASHS is a rare complication of SCA, which often presents as a sudden localized swelling on the scalp with rapid progression. If not thought for, there is a potential for misdiagnosis and may be managed inadvertently through surgical intervention with untoward outcome. The index patient was initially referred to the surgical clinical on the pretext of a concealed traumatic injury. However, upon review the patient was referred to the medical department where a diagnosis of SCA and ASHS were made. This patient, at 16 years of age had not been diagnosed for SCD due to limited access to diagnostic facility. In the majority of health facilities in SSA, sickling test, which has limited predictive value, is usually the available test. This patient tested negative for sickling test at a secondary facility, and thus was never suspected to have SCA despite the subtle clinical presentation of recurrent joint pains and a close family history of SCA in a sibling. Lack of clinical suspicion index among the lower‐cadre healthcare workers poses a serious concern over the quality of healthcare services.^19^ The awareness among healthcare personnel requires immediate redress through on‐job training and continuing medical education which are virtually nonexistent in the rural areas and other resource‐constrained settings in SSA.

This index patient presented with subtle symptoms of recurrent joint pains suggestive of mild VOCs in a mild SCA phenotype. Further laboratory workup revealed a moderate anemia with 9.8 g/dL and microcytic hypochromic anemia. The diagnosis of SCA was confirmed with Hb electrophoresis indicating homozygosity for HbS. This observation of microcytic hypochromic anemia was inconsistent with anemia expected in SCA, however, a recent study by Patel, VP et al demonstrated that while the majority of patients with SCA had sufficient iron levels, a significant proportion were either iron insufficient or had overload.^20^ In fact, it is further argued that iron deficiency in SCD could be much more common than previously thought, and is associated with less tendency of developing VOCs due to lower blood viscosity.^21^ Thus, this index patient could have had concomitant iron deficiency anemia (IDA), unfortunately due to technical limitations the detailed iron studies were not performed in this patient for the confirmation of IDA. Noteworthy, microcytic anemia is a well‐established entity in SCA patients with co‐existing beta thalassemia. The coexistent HbS along with β‐thalassemia that is, compound heterozygotes, HbS‐δβ thalassemia often present with severe symptoms, inconsistent with the index patient, and is usually considered rare in our setting. However, this possibility cannot be ruled out. To better understand this, detailed iron studies along with genetic analysis would be warranted.

Intriguingly, this patient was undiagnosed but remained with mild symptoms without any pharmacologic intervention. Evidence from global SCD molecular and genetic analysis indicates the presence of variable haplotypes across populations, with diverse haplotypic distribution driven by historical migration and genetic drifts. It has been postulated that the diversity of haplotypic variants to a large extent underlies the observed variable expressivity of SCD phenotypes.^1^, ^22^ It appears that specific SCD haplotypes could be associated with yet, unknown modifier genetic variants that could influence the disease phenotype and severity.^1^, ^3^, ^22^, ^23^ These haplotypic and epigenetic diversities have been reported in Western African, Afro‐Arab and Arabic populations. However, these variants have not been characterized in SCD patients of Eastern African Bantu population.^1^, ^24^

Notably, the observed high expression of foetal Hb (HbF) gene, by almost 20% in this patient, which is not often typical of SCA, could partly explain the mild SCA clinical phenotype. It is well established that increased expression of HbF gene in response to hypoxic stimulation helps to offer an alternative high‐affinity oxygen carrier capable of offsetting the typical hypoxemia and subsequent symptomatology. The expression of 15%–40% of HbF typically sets the physiological and biochemical threshold for preventing sickling in SCD.^22^ Thus, the index patients had Hb of 9.8 g/dL and HbF 19.50%, which is comparable to several reported cases, which had Hb levels ranging from 6.0–11 g/gL, and HbS ranging from 65.0%–90.0% respectively and comparable HbF levels.^11^, ^25^, ^26^, ^27^ The expression of HbF and its distribution in RBCs is regulated by elements linked to β‐globulin complex and are associated with specific β‐globin haplotype and *trans*‐acting elements associated with BCL11A and HBS1L‐MYB intergenic region on chromosome 2p16 and 6q23, respectively.^22^ BCL11A is a critical transcriptional factor expressed in hematopoietic tissues which control HbF switching through interaction with GATA1; a DNA‐binding zinc finger motif.^28^, ^29^ This spatial and temporal distribution of HbF partly determines the propensity towards polymerization, sickling, and consequent disease phenotype through inhibition of polymer‐induced damage.^22^

There is a paucity of data on the prevalence and clinical manifestations of patients with ASHS worldwide. However, the available case reports indicates that the majority of patients present with scalp swelling often with involvement of unilateral and seldom bilateral periorbital oedema.^27^ The index patient presented with typical manifestation of ASHS in agreement to other reported cases across countries and ethnicities.^12^, ^30^ There is no any confirmed predilection of specific skull bone, however, of the majority of reported cases, frontal bone appears to be the most commonly affected for reasons which remains largely unknown.^25^ Noteworthy, the available case reports indicates that patients with ASHS are often boys, if not all, with debut typically at puberty, the age range of 11–20 years, the majority being 16 years as the index case.^25^, ^31^, ^32^, ^33^ This stage coincides with the *zenith* of testosterone surge leading to the appreciable secondary male characteristic changes which are associated with increased bone remodeling and growth spurt.^34^ Therefore, it is imperative to speculate the possible influence of testosterone that may exert on bone remodeling, hematopoiesis and increased growth spurt during adolescence, either independently or through enhanced signaling of specific pathways involved in bone remodelling including insulin‐like growth factor 1 (IGF‐1).^34^, ^35^ Thus, in the context of multiple localized skull bone micro‐infarcts, testosterone surge might exert shear stress through bone remodeling leading to the loss of local integrity of bone matrix, weakened cortical bones, and subsequent breakage. Similarly, vitamin D, through binding to the specific nuclear receptors interact with vitamin D response elements (VDRE) on the responsive gene to modulate bone remodeling. This accelerated modulation is postulated to participate in the aetiopathogenesis of ASHS.^36^

In a patient with established diagnosis of SCA, the diagnosis of ASHS can be made clinically. However, it is prudent to undertake brain imaging to rule out any co‐existing ICH for appropriate intervention. The index patient was initially seen at a secondary facility where CT scan services were not available. Lack of advanced brain imaging services in SSA is major hurdle in managing patients where ICH is of potential concern. However, in the absence of ICH, complete resolution of ASHS is the rule, on conservative management.^30^, ^33^, ^37^ Thus, a high index of suspicion for ASHS is critical, particularly where advanced diagnostic services are not readily available. Nevertheless, patients with initial episode of ASHS are at increased risk of recurrency and ICH, thus, patients would still require long‐term follow up. Early diagnosis of SCD, regular clinic visits with well‐staffed and equipped facility, optimized care on supplemental folic acid and hydroxyurea administration are the cornerstone to the prevention of complications of SCA including ASHS. The goal should be the attainment of Hb of about 8 g/dL, and prevention of VOCs.^38^

## 4 CONCLUSION

Spontaneous SGH is a rare clinical entity, with sporadic cases reported as a complication of SCD. It is prudent for the clinicians to beware of ASHS, particularly in the resource‐limited setting, where imaging services are not readily accessible. Though most cases resolve with conservative measures, this condition could be associated with life‐threatening ICH.

## AUTHOR CONTRIBUTIONS

**Ng'weina F. Magitta:** Conceptualization; investigation; writing – original draft; writing – review and editing. **Francisca B. Komanya:** Conceptualization; investigation; writing – review and editing. **Baraka O. Alphonce:** Conceptualization; investigation; writing – review and editing. **Mbelwa D. Bitesigilwe:** Investigation; visualization; writing – review and editing. **Emmanuel M. Sindato:** Conceptualization; supervision; writing – review and editing. **John R Meda:** Conceptualization; supervision; writing – review and editing.

## FUNDING INFORMATION

There was no fund for this study.

## CONFLICT OF INTEREST STATEMENT

The authors declares no conflict of interest.

## ETHICS STATEMENT

This case report did not require ethics approval.

## CONSENT

The patient's guardian consented in writing for the publication of this report. The written and signed consent form is available to the Editor‐in‐Chief if required.

## Data Availability

The raw data pertaining to this case report is available on reasonable request.
